# Dural Tear and Cerebrospinal Fluid Leakage in Anterior Cervical Spine Surgery: Pathophysiology, Management, and Evolving Repair Techniques

**DOI:** 10.3390/jcm14238478

**Published:** 2025-11-29

**Authors:** Jae Jun Yang, Jiwon Park, Jong-Beom Park, Suo Kim

**Affiliations:** 1 Department of Orthopaedic Surgery, Dongguk University Ilsan Hospital, Ilsan 10326, Republic of Korea; jaejunyang@gmail.com; 2 Department of Orthopaedic Surgery, Korea University Ansan Hospital, Ansan 15328, Republic of Korea; jwpark506@gmail.com; 3 Department of Orthopaedic Surgery, Uijeongbu St. Mary’s Hospital, College of Medicine, The Catholic University of Korea, Uijeongbu 11765, Republic of Korea; kimsuo3897@gmail.com

**Keywords:** dural tear, cerebrospinal fluid leak, ossification of posterior longitudinal ligament, anterior cervical spine surgery, dural repair, sealants, lumbar drainage

## Abstract

Dural tear (DT) and cerebrospinal fluid (CSF) leakage, though uncommon complications, represent a potentially serious risk of anterior cervical spine surgery, particularly in patients with ossification of the posterior longitudinal ligament (OPLL). While the incidence in routine anterior cervical discectomy and fusion (ACDF) or corpectomy (ACCF) is typically below 0.5%, it rises sharply to 4–32% in OPLL cases. Furthermore, it exceeds 60% when dural ossification (DO) is present. Adhesion and ossification obliterate the normal epidural plane, creating a fragile osteofibrotic interface that is highly susceptible to tearing during decompression. This review synthesizes current evidence on the pathophysiology of DT and CSF leakage in anterior cervical spine surgery, provides a framework for risk stratification, and outlines evolving techniques for successful repair and management. Intraoperative management has shifted from direct resection toward dura-preserving floating decompression and biologically reinforced multilayer repair using fascia, collagen matrix, fibrin adhesives, and polyethylene glycol (PEG) hydrogel sealants. Postoperative care emphasizes controlled CSF pressure regulation, sterile wound management, and early ambulation. Most DTs achieve successful closure with timely recognition and standardized treatment. However, persistent leakage may require escalation to composite reconstruction, epidural blood patch, or vascularized flap reinforcement. Emerging technologies such as bioactive hydrogels, 3D-printed dural scaffolds, and artificial intelligence–assisted imaging offer potential future improvements, although clinical adoption remains limited. This review summarizes current evidence on the mechanisms, risk factors, diagnostic predictors, repair strategies, and postoperative management of DT and CSF leakage, with specific attention to OPLL-related DO. A more apparent distinction between established clinical practice and emerging investigational technologies is provided to guide evidence-based decision-making.

## 1. Introduction

Dural tear (DT) and cerebrospinal fluid (CSF) leakage are uncommon but clinically important complications of anterior cervical spine surgery [1,2]. In routine anterior cervical discectomy and fusion (ACDF) or anterior cervical corpectomy and fusion (ACCF), their incidence remains low (<0.5%) [3,4]. However, the risk increases substantially in surgeries for ossification of the posterior longitudinal ligament (OPLL), where dural adhesion and dural ossification (DO) markedly alter the standard epidural interface [5,6,7,8]. OPLL frequently forms a rigid osteofibrotic complex in which the PLL and dura become tightly fused, resulting in loss of elasticity and a high susceptibility to tearing during decompression [7,8,9]. Even minor dural defects can result in persistent leakage due to the confined anterior cervical corridor and continuous pulsatile CSF pressure [8,10].

**Figure 1:**
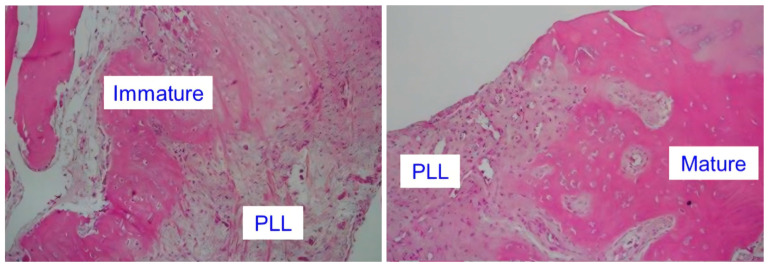
Histology showed an ossification process consisting of immature and mature OPLL.

Subsequent radiologic and histopathologic studies have demonstrated that OPLL is not merely a calcified ligament but a dynamic ossification process involving osteogenic signaling, inflammatory changes, and microstructural remodeling at the dura–PLL junction (Figure 1) [4,5,8,11]. Modern imaging techniques have significantly improved preoperative recognition of high-risk anatomy. Radiologic predictors such as the double-layer sign [9,12,13], hook sign [14,15], a broad OPLL base exceeding 60% [14,15], and K-line negativity [5] correlate strongly with DO and are associated with increased risk of intraoperative DT. Three-dimensional CT reconstruction further enhances visualization of the ossified dura interface and assists in planning safer decompression trajectories [16].

Although OPLL accounts for most DTs encountered during anterior cervical surgery, other etiologies also merit acknowledgment. Trauma, revision surgery, chronic steroid exposure, and prior infection can compromise dural elasticity or create dense adhesions, thereby increasing susceptibility to accidental tearing during anterior decompression [17,18,19,20]. These factors are important to consider during preoperative planning, even when OPLL is not present.

Historically, anterior decompression for OPLL emphasized direct resection of the ossified mass. However, this approach was associated with high DT rates and considerable morbidity, with early studies reporting DT incidences up to 30% [2,21]. Recognition of these complications led to the development of the floating decompression technique, in which the ossified mass is thinned circumferentially and allowed to “float” while preserving the adherent dura [22,23,24]. This strategy significantly reduced leakage rates and shifted the operative paradigm from aggressive resection to dura-preserving decompression [12,25].

As the number of anterior cervical procedures increases globally and the prevalence of OPLL rises with aging populations [26], DT and CSF leakage have become complications of growing clinical and socioeconomic significance. Persistent leakage can prolong hospitalization, increase infection risk, lead to pseudomeningocele formation, or necessitate reoperation [10,18,27]. Nevertheless, when promptly recognized and managed with structured, multidisciplinary approaches—such as multilayer biological closure, collagen or fascia grafts, and controlled lumbar drainage—successful outcomes exceeding 90% have been reported, with neurological recovery comparable to that in cases without DT [26,27,28].

This review synthesizes current evidence on epidemiology, pathophysiology, radiologic risk stratification, intraoperative repair strategies, and postoperative management of DT and CSF leakage after anterior cervical surgery. Particular emphasis is placed on OPLL-associated DO—while clearly distinguishing findings applicable to general cervical procedures—and the review also discusses emerging technologies such as bioactive sealants, 3D-printed scaffolds, and artificial intelligence–assisted imaging, clarifying their current level of clinical maturity and limitations [20,29,30,31,32,33]. This narrative review was conducted using a targeted literature search of PubMed, Embase, Scopus, and Google Scholar. Keywords included “dural tear,” “cerebrospinal fluid leak,” “anterior cervical spine surgery,” “OPLL,” “dural ossification,” and “dural repair.” Priority was given to studies published between 2000 and 2025, with additional inclusion of seminal earlier works when historically relevant. Reference lists of key articles were manually screened to identify additional pertinent publications. Because this review synthesizes heterogeneous clinical and preclinical evidence, no formal systematic review methods or meta-analytic techniques were applied.

## 2. Epidemiology and Risk Factors

### 2.1. Incidence Across Surgical Contexts

The incidence of DT during anterior cervical spine surgery varies widely depending on underlying pathology and approach. In routine degenerative procedures such as ACDF or ACCF, DT is relatively rare, typically occurring in only 0.2–0.5% of cases [3,4,18,27]. In contrast, cases involving OPLL demonstrate markedly higher incidences, mainly due to adhesion between the PLL and dura and the presence of DO. Multiple studies report DT rates ranging from 4 to 32% in OPLL [5,6,7,8,9], and more than 60% when DO is radiologically confirmed [7,8]. Yu et al. reported DT in 63.6% of patients with DO, compared with 3.5% without DO [5], highlighting DO as the most significant determinant of DT risk (Table 1).

### 2.2. Influence of Surgical Approach

The surgical approach strongly influences DT risk. A 2016 meta-analysis reported a DT incidence of 31% for anterior decompression versus 9.3% for posterior surgery (odds ratio 1.9) [17]. Anterior surgery requires direct manipulation of the ossified PLL–dura interface and offers limited working space, thereby increasing the likelihood of DT during drilling or decompression [4,22]. Although posterior decompression is preferred by many experts for continuous or massive OPLL, anterior surgery remains essential in cases with significant ventral compression, a negative K-line, or kyphotic deformity where posterior-only decompression is insufficient [5,27].

### 2.3. Patient-Related Risk Factors

Several patient-related characteristics have been associated with increased DT risk. Advanced age (>65 years) is associated with diminished dural elasticity and impaired wound healing [18,30]. Obesity (BMI ≥ 30) increases epidural venous pressure and challenges visualization [34]. Chronic steroid use weakens collagen structure and delays repair [35]. Revision surgery is another consistent risk factor because scar tissue obliterates regular tissue planes and creates dense adhesions [17,19,20]. Preoperative optimization—including glycemic control, nutritional support, and tapering corticosteroids—is therefore recommended [36].

### 2.4. Radiologic Predictors and Morphological Correlates

**Figure 2:**
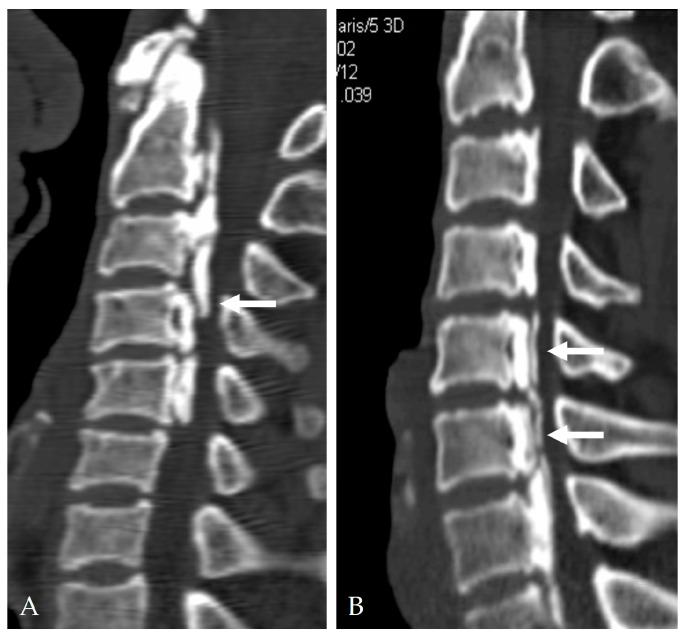
2-dimensional sagittal reconstructed CT showed ‘double-layer’ sign (white arrows) (**A**,**B**).

**Figure 3:**
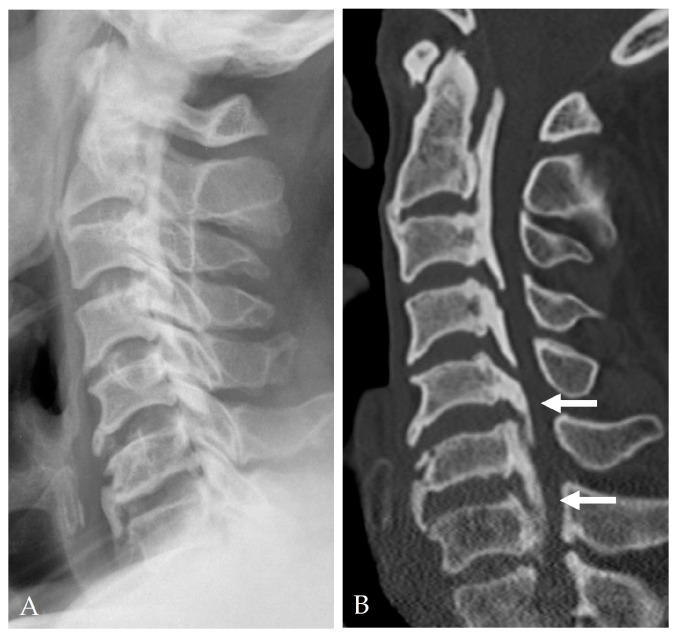
Lateral X-ray and 2-dimensional sagittal reconstructed CT showed ‘negative K-line’ OPLL (white arrows) (**A**,**B**).

High-resolution CT and MRI play essential roles in preoperative risk assessment. Several radiologic signs strongly correlate with DT:

- Double-layer sign: Indicates DO and consists of two dense rims separated by a radiolucent area (Figure 2) [9,12,13].
- Hook sign: Suggests focal bony penetration into the dura or severe adhesion [14,15].
- OPLL occupying ratio ≥ 60%: Associated with firm dural adherence [14,15].
- Negative K-line: Reflects anterior cord compression and a higher risk during anterior decompression (Figure 3) [5].
- Broad-based or continuous/mixed-type OPLL: Confers approximately 10-fold higher risk of DT than segmental types [37].

A machine–learning–based CT scoring system developed by Du et al. incorporated these predictors and demonstrated an AUC of 0.94, outperforming manual interpretation [16]. These parameters are now integral to surgical planning.

### 2.5. Disease-Specific Factors: Dural Ossification and Adhesion Severity

DO is the most important disease-specific determinant of DT. DO fundamentally alters the mechanical properties of the dura, reducing flexibility and introducing microscopic channels that communicate with the subarachnoid space [7,8]. Histologic studies confirm that ossified dura contains bone marrow elements and neovascular channels, making it more fragile under drilling forces [9,12]. Severe adhesion between the OPLL and dura leads to direct stress transmission during decompression, often exceeding physiological shear thresholds demonstrated in ex vivo and finite element (FE) models [16,27].

### 2.6. Intraoperative Technical Factors

Technical aspects significantly influence DT occurrence. Aggressive resection of adherent OPLL, deep or uncontrolled drilling, and thermal injury from burrs increase the likelihood of tearing [12,22,23]. Inadequate visualization in narrow corridors contributes to accidental penetration. Conversely, the use of operative microscopy or endoscopic assistance improves visualization and reduces unwanted traction on the dura [38]. Indirect decompression techniques—particularly floating decompression—mitigate dural tension and lower DT incidence [24,25].

### 2.7. Clinical Consequences and Prognostic Significance

Although DT increases operative duration, blood loss, and hospital stay [27], timely repair typically prevents long-term neurological deterioration. Du et al. reported complete resolution of all DTs in their series of anterior OPLL surgeries using fascia onlay and fibrin sealant reinforcement [16]. Across studies, over 90% of patients achieve satisfactory outcomes when leaks are recognized early and treated appropriately [18,26,28]. However, inadequately treated leaks can lead to pseudomeningocele, infection, intracranial hypotension, or reoperation—risks that rise as high as fivefold in uncontrolled leakage [10,18,27].

### 2.8. Summary

In summary, DT incidence is low in routine ACDF/ACCF but rises sharply in OPLL, particularly with DO. Anterior decompression carries a higher risk than posterior approaches due to direct manipulation of the ossified dura–PLL complex. Radiologic predictors—including the double-layer sign, hook sign, negative K-line, and a broad OPLL base—provide strong preoperative risk stratification. Patient factors such as age, obesity, steroid exposure, and revision surgery further modify risk. A comprehensive understanding of these variables is essential for appropriate surgical planning and for mitigating CSF leakage risk [3,4,7,8,16,27].

## 3. Pathophysiology of Dural Tear and Cerebrospinal Fluid Leakage

The pathophysiology of DT and CSF leakage during anterior cervical surgery is primarily influenced by the degree of adhesion, ossification, and mechanical stress along the PLL–dura interface. These changes are particularly prominent in OPLL, where progressive ossification eliminates the normal epidural plane and produces a structurally vulnerable dural surface (Table 2) [3,4,7,13].

### 3.1. Microstructural and Cellular Alterations of the PLL–Dura Interface

OPLL is now recognized as an active endochondral ossification process rather than a passive degenerative change. Histopathologic studies demonstrate that fibroblasts within the PLL–dura junction undergo osteogenic metaplasia and express bone morphogenetic protein (BMP-2), runt-related transcription factor 2 (Runx2), and vascular endothelial growth factor (VEGF) [3,4,11,26,39]. Macrophage infiltration and neovascular ingrowth further weaken tissue organization, while matrix metalloproteinase (MMP) activity contributes to extracellular matrix remodeling [26,39]. Electron microscopy demonstrates fragmentation of type I and III collagen fibers and loss of elastic fiber continuity, resulting in reduced tensile integrity of the dura [4,11]. These cellular and microstructural changes explain why even minimal manipulation during anterior decompression may convert microscopic weaknesses into full-thickness tears, particularly when DO is present [8,9,10,22].

### 3.2. Biomechanical Transformation of the Ossified Interface

Under normal circumstances, the epidural space acts as a biomechanical buffer, distributing shear forces during cervical motion. In OPLL, ossified fusion between the PLL and dura abolishes this buffer, transmitting drilling forces directly to the dural surface [8,9]. Ex vivo mechanical testing and FE analyses demonstrate that shear strain at ossified junctions can exceed 12–15%, surpassing the approximate yield threshold of healthy dura (~5%) [19,24]. This mechanical vulnerability is amplified in DO, where the dura directly incorporates bony trabeculae and marrow channels [7,8]. Intraoperative maneuvers—intense drilling, thermal injury from burrs, or traction at adhesion margins—may therefore lead to rapid propagation of minor defects, underscoring the need for meticulous micro-drilling and dura-preserving techniques [22,23,24].

### 3.3. Role of Dural Ossification (DO) in Leak Formation

DO represents the strongest disease-specific risk factor for DT. Radiologic and histologic analyses show that DO may contain intradural bone marrow, neovascular channels, and irregular microfractures [9,12], all of which compromise the mechanical resilience of the dura. Even slight oscillatory pressure from surgical instruments can disrupt this fragile structure. Yu et al. found DT in over 60% of DO-positive cases, compared to rates below 4% in OPLL without DO [7]. Because DO directly influences structural behavior under stress, its presence should guide the selection of indirect decompression methods such as floating decompression [24,25].

### 3.4. CSF Pressure Dynamics and Enlargement of Microdefects

Once a dural defect forms, CSF egress is driven by the pressure gradient between the subarachnoid and prevertebral spaces. The anterior cervical corridor is narrow and offers limited soft-tissue resistance; therefore, CSF rapidly accumulates subfascially, promoting pseudomeningocele formation [18,27]. Physiologic CSF pulsation (typically 8–12 mmHg) progressively enlarges microdefects, a mechanism supported by experimental in vitro and animal studies demonstrating delayed collagen deposition in CSF-exposed tissue [18,19].

Because CSF contains low fibrinogen levels and high MMP activity, clot formation is impaired, and fibroblast proliferation is reduced by up to 40% in CSF-rich environments [19,39]. This biochemical milieu delays spontaneous dural closure, underscoring the importance of postoperative pressure regulation.

### 3.5. Inflammatory and Biochemical Factors

DT initiates an inflammatory cascade involving TNF-α, IL-1β, and other mediators that increase vascular permeability and degrade extracellular matrix components [39]. Prolonged inflammation delays wound healing, while excessive fibroplasia may lead to arachnoid scarring and adhesions. In patients with diabetes, chronic steroid use, or systemic illness, these inflammatory effects are potentiated, further impairing dural regeneration [35]. Additionally, chronic CSF leakage creates an osmotic gradient that draws interstitial fluid into the cavity, increasing pseudomeningocele size and creating a self-perpetuating cycle of leakage and delayed healing [18].

### 3.6. Arachnoid Integrity as a Determinant of Leak Severity

Preservation of the arachnoid membrane plays a central role in limiting CSF flow after DT. Studies indicate that when the arachnoid remains intact—despite outer dural disruptions—CSF egress is significantly reduced, and the likelihood of persistent fistula formation is markedly lower [24,25]. In contrast, combined arachnoid-dural penetration establishes direct communication with the surgical field, facilitating continuous leakage and increasing infection risk [28,40].

### 3.7. Secondary Sequelae and Systemic Consequences

If uncontrolled, CSF leakage may progress through stages: subfascial accumulation, pseudomeningocele formation, intracranial hypotension, and, in severe cases, meningitis or sepsis [18,27,41,42]. Clinical manifestations include orthostatic headache, nausea, cranial nerve symptoms, and wound dehiscence. Rare but important complications, such as remote cerebral hemorrhage, have been reported and should be considered in postoperative neurological decline [43].

### 3.8. Summary of Pathophysiology of Dural Tear and Cerebrospinal Fluid Leakage

The development of DT and CSF leakage in anterior cervical surgery reflects a combination of cellular degeneration, biomechanical vulnerability related to ossification, and adverse CSF pressure dynamics. OPLL—especially with DO—eliminates the standard epidural buffer, allowing surgical forces to directly impact a structurally fragile dura [7,8,9]. Mechanical and pulsatile stresses enlarge even microscopic defects, while CSF biochemistry and inflammation delay closure [19,39]. Understanding these factors is essential for selecting preventive strategies such as floating decompression, implementing multilayer biological sealing, and ensuring effective postoperative pressure control [24,25,28].

## 4. Intraoperative Repair Technique

Effective intraoperative management of DTs during anterior cervical surgery requires a structured, size-based, and biologically reinforced strategy because the anterior corridor limits suturing and direct manipulation [12,24,25]. Most techniques rely on multilayer sealing, autologous or collagen-based graft reinforcement, and controlled CSF pressure management to ensure durable closure (Table 3) [16,19,27].

### 4.1. Classification and Principles of Repair

Epstein’s size-based classification—small (<5 mm), moderate (5–10 mm), and large (>10 mm) defects—remains widely used in clinical decision-making [14].

**Figure 4:**
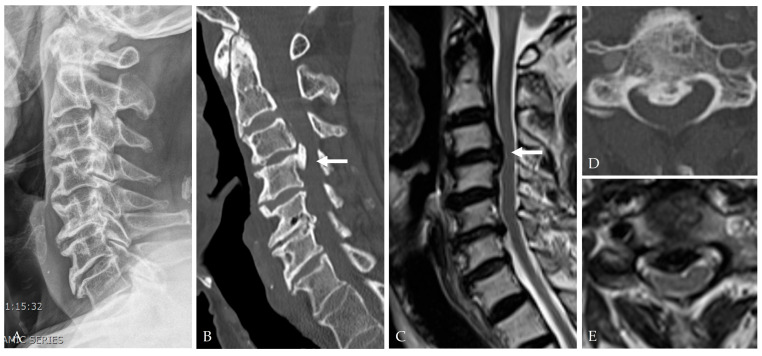
A 79-year-old male patient complained of cervical myelopathy. Latreal X-ray (**A**), CT (**B**,**D**), and MRI (**C**,**E**) showed C3–4 OPLL compressing the spinal cord (white arrow).

**Figure 5:**
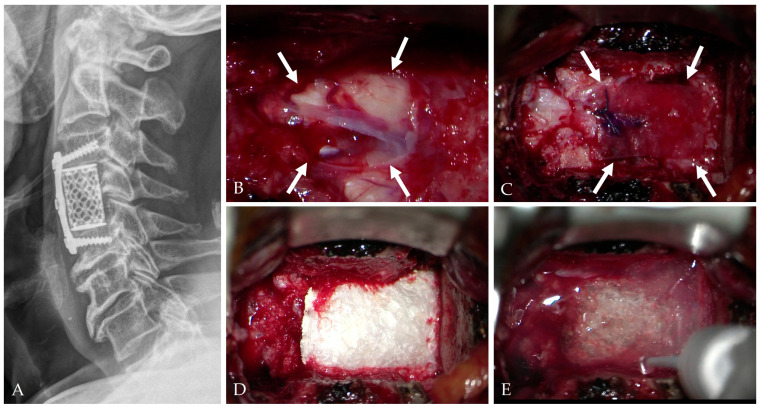
The patient underwent C3–5 ACCF (**A**). During the surgery, dura tear and CSF leakage occurred (white arrows) (**B**) and were managed with artificial dura (white arrows) (**C**), Tacocom (**D**), and sealant (**E**).

**Figure 6:**
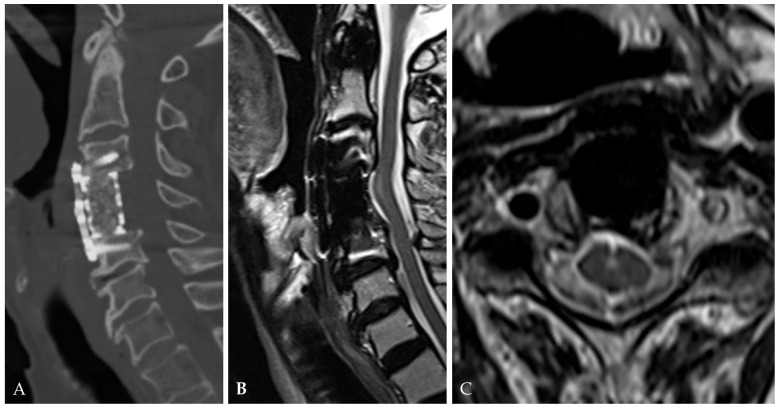
At postoperative 1 month, CT (**A**) and MRI (**B**,**C**) showed complete decompression of the C4–5 OPLL and healing of the dura tear and CSF leakage.

- Small defects are typically managed with gelatin sponge reinforcement and short-term lumbar drainage [28,44].
- Moderate defects benefit from fascia or pericardium onlay reinforced with fibrin or polyethylene glycol (PEG) hydrogel sealants, which improve conformity and watertightness [19,25,45].
- Large defects often require composite multilayer constructs combining artificial dura, sealant, and fat graft to restore mechanical stability and minimize CSF pulsation (Figure 4, Figure 5 and Figure 6) [25,26,29].

In OPLL with DO, where primary suturing is rarely feasible, indirect reinforcement and multilayer biological reconstruction are essential because the residual dura is fragile and often irregular [7,9,12].

### 4.2. Floating Repair and Dura-Preserving Techniques

The floating decompression technique—first described by Lei and colleagues—reduces dural traction by thinning the ossified mass circumferentially while preserving the adherent dura [24]. This method avoids direct manipulation of the ossified dural interface and markedly reduces DT risk and severity, with reported leakage rates dropping from ~28% to below 10% in contemporary series [24,25,28]. Floating repair is beneficial when DO is present, as direct removal carries a significantly higher risk of rupture [9,12,22,23].

### 4.3. Autologous Grafts and Biological Reinforcement

**Figure 7:**
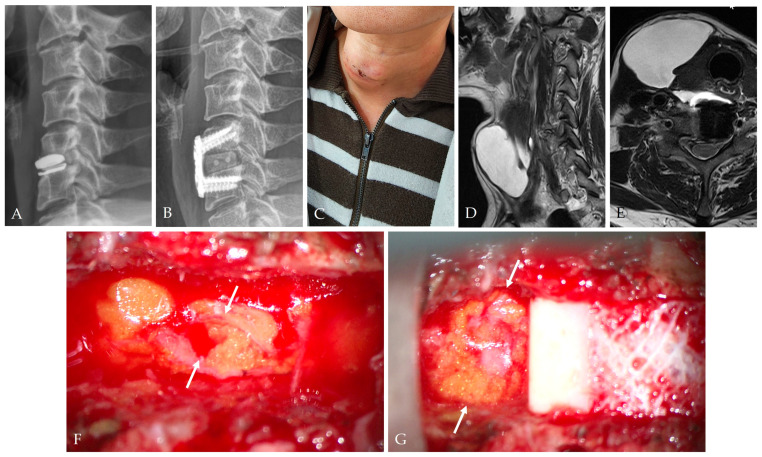
A 51-year-old female patient underwent C5–6 ACDF for failure of cervical disc arthroplasty (CDA) (**A**,**B**). At postoperative 2 weeks, the patient complained of anterior right side neck swelling (**C**). Follow-up MRI showed an extensive CSF collection (**D**,**E**). During the revision surgery, a dural tear (white arrows) was identified (**F**), and fat graft (white arrows) was applied to close the CSF leakage site (**G**).

Autologous grafts (fascia lata, temporalis fascia, fat) remain the foundation of biological closure:

- Fascia onlay patches provide tensile strength and promote fibroblast ingrowth [44,45].
- Fat grafts dampen CSF pulsation and promote granulation tissue formation (Figure 7) [19,26].

Clinical studies using fascia onlay plus fibrin reinforcement report > 95% closure success, although these outcomes arise from small series and should be interpreted cautiously [14,28].

### 4.4. Artificial Dural Substitutes

Collagen-based dural matrices (e.g., DuraGen^®^, AlloDerm^®^) act as resorbable scaffolds that integrate within 6–8 weeks and minimize inflammatory response [20,29]. Experimental data suggest near-complete neodural formation by two months in animal models, but high-quality comparative clinical trials remain limited [20,29,30].

### 4.5. Sealants and Adhesives

Sealants improve watertightness and are widely used in the anterior cervical corridor:

- Fibrin sealants (Tisseel^®^, Evicel^®^) reinforce fascia and achieve burst pressures around 120 mmHg in ex vivo assays [25,26].
- PEG hydrogels (Duraseal^®^, Liqoseal^®^) offer elastic sealing and controlled degradation [19].
- Fibrin-coated collagen patches (TachoSil^®^) have demonstrated excellent adherence, even in confined spaces, with Gazzeri et al. reporting only one recurrence in 35 patients [26].

However, sealants alone rarely suffice for DO-related defects and should be combined with graft materials for stability [12,25,29].

### 4.6. Composite “Sandwich” Repair

Composite multilayer “sandwich” constructs integrate artificial dura, hydrogel, or fibrin sealant, and an autologous fat or fascia layer to produce immediate mechanical reinforcement and biological integration [19,25,44,45]. PGA mesh with fibrin reinforcement has demonstrated complete leak resolution in small cervical series, though evidence remains limited to observational reports [28,29].

### 4.7. Controlled CSF Diversion

Post-repair pressure regulation is critical. Traditional gravity-dependent lumbar drains risk both under- and over-drainage [19,38]. In contrast, pump-regulated volumetric continuous lumbar drainage (PRVCLD) provides precise flow control (5–10 mL/h) at 6–8 cm H_2_O and has shown > 90% resolution of leaks without meningitis in a 2023 study [28]. Alternate CSF diversion methods (ventriculostomy, wound–peritoneal shunts) may be necessary when lumbar access is not possible [19,30,44].

### 4.8. Vascularized Flap Reinforcement

For recurrent or refractory DTs, vascularized flaps such as sternocleidomastoid (SCM) or pectoralis major can provide robust coverage and enhance healing by augmenting blood supply [19,26]. These flaps are reserved for revision procedures or irradiated/compromised soft-tissue beds.

### 4.9. Summary of Intraoperative Repair Technique

A structured, size-based, multilayer approach remains the most effective strategy for DT closure in anterior cervical surgery. Autologous grafts, collagen matrices, sealants, and controlled CSF diversion together reduce persistent leak rates to <5% in most series [26,28,44].

## 5. Postoperative Management and Outcomes

Postoperative management critically determines the durability of dural repair, as even technically successful closures may fail without proper pressure control, wound care, and early detection of recurrence (Table 4) [18,19].

### 5.1. CSF Pressure Regulation

**Figure 8:**
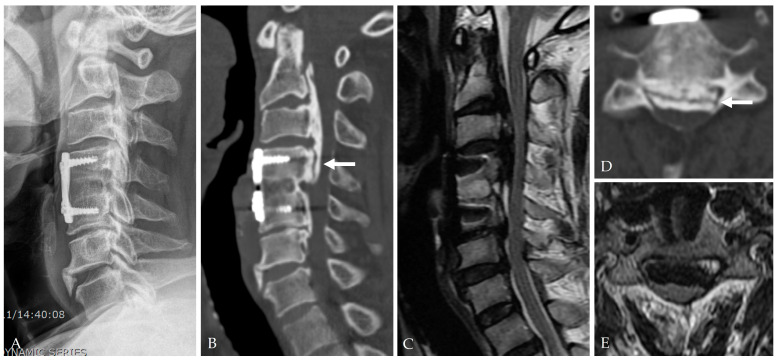
A 66-year-old male patient complained of cervical myelopathy. He underwent C4–5 ACDF 22 years ago. Latreal X-ray (**A**), CT (**B**,**D**), and MRI (**C**,**E**) showed C2–7 OPLL compressing the spinal cord and ‘double-layer’ sign (white arrow).

**Figure 9:**
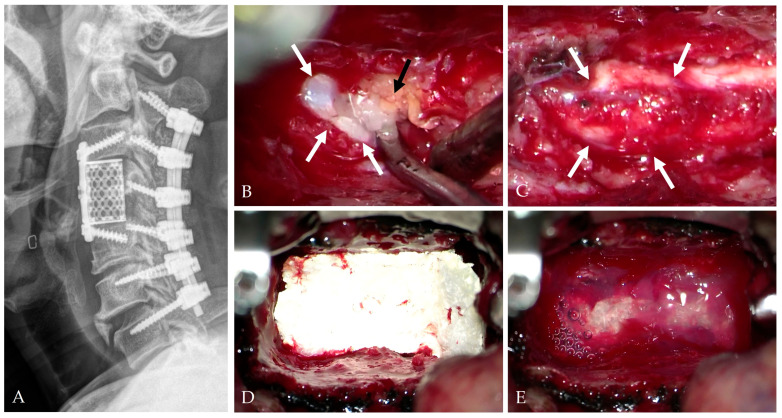
The patient underwent C2–7 laminectomy and fusion and C3–5 ACCD (**A**). During the anterior surgery, a dural tear and CSF leakage (white arrows) occurred due to dural ossification with OPLL (dark arrow) (**B**,**C**), which were managed with Tacocom (**D**) and sealant (**E**).

**Figure 10:**
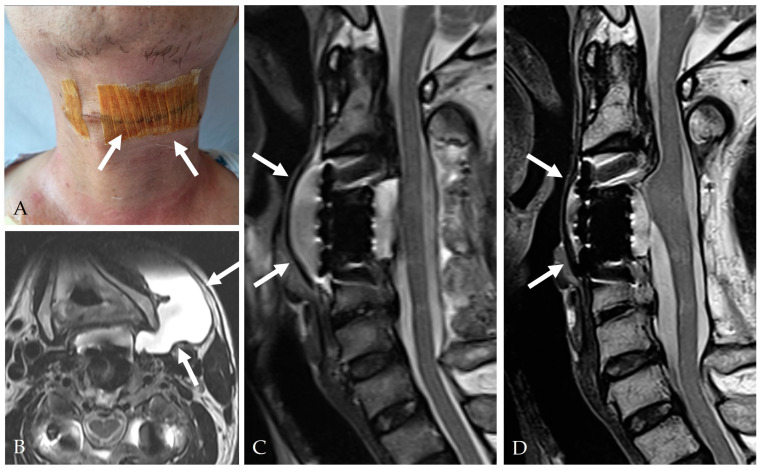
At postoperative 13 days, the patient complained of anterior left side neck swelling (**A**). Follow-up MRI showed an extensive CSF collection (**B**,**C**). Revision surgery was performed for CSF drainage and augmented closure of the CSF leakage site with Tacocom and sealant. In addition, lumbar drainage was performed for 5 days. One month after revision surgery, follow-up MRI showed healing of the dural tear and a small remnant of CSF (**D**).

Controlled lumbar drainage remains the cornerstone of postoperative care. PRVCLD offers stable intracranial and spinal CSF pressure settings and permits early mobilization with low infection rates [28]. Traditional gravity-based drainage systems are more prone to over-drainage and pose a higher risk of complications (Figure 8, Figure 9 and Figure 10) [19,38].

### 5.2. Positioning and Mobilization

Neutral or slight-lateral positioning for 48–72 h minimizes anterior hydrostatic pressure on the repair site [10,27]. Ambulation is typically safe once drain output decreases to <30–50 mL/day [19,41]. Early mobilization improves pulmonary outcomes and reduces the risk of thromboembolism without increasing leak recurrence [28,43].

### 5.3. Wound Care and Infection Prevention

Because CSF inhibits normal collagen synthesis, wound healing is often delayed. Sterile dressings, daily inspection, and 5–7 days of prophylactic antibiotics (e.g., cefazolin ± gentamicin) are recommended [33]. Persistent drainage (>7 days) may indicate superficial dehiscence requiring revision or fat graft reinforcement [19,32,46,47,48].

### 5.4. Pseudomeningocele Management

Pseudomeningoceles occur in up to 30% of cases with DT [18,33,43].

- <2 cm → observation
- 2–4 cm → aspiration and compression
- 4 cm or symptomatic → operative obliteration with fat or muscle grafts [19,26]

Most pseudomeningoceles resolve spontaneously within several weeks; recurrence typically suggests residual DO or inadequate drainage [25,28,49,50,51,52].

### 5.5. Adjunctive Therapies

Epidural blood patch (EBP) provides effective tamponade, with success rates exceeding 80% in postoperative spinal leaks [44,45]. Negative-pressure wound therapy and biologic sealant injection may also be used to supplement management in selected patients [18].

### 5.6. Functional Outcomes

With early recognition and standardized protocols, >90% of patients achieve complete resolution of leakage and neurological outcomes similar to those of patients without DT [18,26,27,28]. Unrecognized or inadequately treated leaks significantly increase reoperation rates (OR ~5) [27].

## 6. Complications and Reoperation

Although modern repair techniques have improved outcomes, persistent CSF leakage may lead to local, systemic, and mechanical complications requiring early intervention.

### 6.1. Early Local Complications

Subfascial CSF accumulation and wound swelling occur in up to 30–40% of OPLL cases with intraoperative leakage [18,19]. Progressive swelling suggests persistent leakage and necessitates re-exploration or reinforcement [25,28]. Durocutaneous fistula formation provides a direct route for infection and warrants immediate closure [53].

### 6.2. Infectious Complications

Although overall infection rates are low (0.5–2%), CSF leakage increases the risk due to impaired local immune defenses [18,33]. Cases of meningitis—including those due to *Klebsiella pneumoniae*—have been reported, requiring intravenous and/or intrathecal antibiotics [33].

### 6.3. Intracranial Hypotension and Remote Cerebral Hemorrhage

Persistent drainage or high-output leaks may lead to intracranial hypotension characterized by orthostatic headache, nausea, diplopia, and pachymeningeal enhancement on imaging [27,43]. Remote cerebral hemorrhage, though rare, is an important complication associated with excessive CSF loss and should be considered in patients with neurological decline [43].

### 6.4. Mechanical/Implant-Related Complications

Implant failure and graft subsidence may occur due to altered biomechanics and non-union after anterior OPLL surgery [27]. A meta-analysis showed that hardware failure increases the risk of reoperation by more than 190-fold (OR 192.09) [10,27]. Combined anterior–posterior stabilization is often required in revision cases.

### 6.5. Pseudomeningocele and Late Recurrence

Most small pseudomeningoceles resolve conservatively [33,43]. Recurrence indicates persistent dural defects, incomplete closure, or unresolved DO fragments requiring imaging evaluation and possibly revision surgery [16,18,28].

### 6.6. Reoperation Rates and Predictors

Overall reoperation rates after OPLL surgery approximate 5% [27,37].

Strong predictors include:

- DT occurrence (OR 4.97)
- Kyphosis or loss of lordosis < 10°
- Hardware failure (OR 192.09) [27]

### 6.7. Summary of Complications and Reoperation

Modern biologically based repair strategies and regulated drainage systems have reduced long-term complications. Nevertheless, persistent leakage, infection, intracranial hypotension, and hardware failure remain important considerations that must be addressed through standardized protocols [26,28,44].

## 7. Future Directions and Technological Innovations

### 7.1. Overview

Despite progress in dural repair, current methods remain primarily mechanical. Future developments aim to promote proper biological regeneration while enhancing safety and predictability [20,29,30,31]. However, most innovations remain experimental, and high-quality clinical data are limited.

### 7.2. Bioactive Hydrogels

Next-generation hydrogels incorporating alginate, chitosan, or collagen demonstrate improved burst pressures (>200 mmHg) and sustained antimicrobial or growth-factor release in preclinical models [20,31]. Their ability to support fibroblast migration and angiogenesis has been demonstrated in small animal studies, but human data are lacking.

### 7.3. 3D-Printed Bioresorbable Scaffolds

3D printing enables the fabrication of patient-specific dural scaffolds from polycaprolactone (PCL) and gelatin composites [30]. These constructs replicate native collagen alignment and have shown promising results in animal models for neo-ductal formation. Clinical translation has not yet been established.

### 7.4. Regenerative Medicine and Stem Cells

Mesenchymal stem cell (MSC)–seeded membranes promote collagen deposition and faster dural remodeling in preclinical studies [20,31]. Exosome-loaded hydrogels may further enhance healing while avoiding issues associated with live-cell transplantation. These remain investigational and require rigorous safety evaluation.

### 7.5. Nanotechnology and Biosensing Dura

Nanoparticle-enhanced membranes (silver, zinc, graphene oxide) offer antimicrobial protection and improved mechanical properties in vitro [20,30]. A biosensing “smart dura” capable of detecting pH or enzymatic markers of inflammation is under development but has no clinical application yet [31].

### 7.6. Artificial Intelligence (AI)

AI-enhanced CT interpretation improves the detection of DO and other high-risk features, achieving AUC values up to 0.94 in recent studies [16]. AI-assisted navigation and augmented reality tools may one day guide safer decompression, although these technologies remain developmental and require validation in prospective clinical trials [31].

### 7.7. Standardization and Research Needs

A significant challenge is the absence of standardized definitions and outcome metrics—27 different definitions of CSF leak have been reported across studies [33]. Multicenter registries run by organizations such as CSRS-AP and AO Spine could provide high-quality datasets for comparative analysis and machine learning.

### 7.8. Summary of Future Directions and Technological Innovations 

Future progress will rely on integrating biomaterials science, regenerative medicine, and AI-driven diagnostics into clinically feasible strategies. Most emerging technologies remain at an early experimental stage, and robust clinical trials are needed before widespread adoption [31,32,33].

## 8. Limitations

This review is limited by the heterogeneity of existing studies, which differ in definitions of CSF leakage, diagnostic criteria, and postoperative outcome reporting, reducing cross-study comparability. Much of the available evidence—especially regarding OPLL and DO—relies on small, retrospective cohorts, and emerging repair technologies remain supported largely by preclinical or early feasibility data. High-quality, prospective multicenter studies with standardized metrics are needed to validate current strategies and strengthen evidence-based recommendations.

## 9. Conclusions

DT and CSF leakage remain clinically significant complications of anterior cervical spine surgery, especially in patients with OPLL and DO. Although rare in routine ACDF or ACCF, OPLL-induced adhesion and ossification substantially increase the risk of intraoperative rupture. Advances in CT, MRI, and AI-assisted assessment now improve the preoperative identification of high-risk anatomy and support more individualized surgical planning.

Modern management emphasizes durable, floating decompression and reinforced, multilayer biological repair using autologous grafts, collagen matrices, and fibrin- or PEG-based sealants. Standardized postoperative care—including controlled CSF diversion, wound surveillance, and timely mobilization—further enhances repair durability. When promptly recognized and managed with protocol-driven strategies, most DTs resolve without long-term neurological consequences.

Recurrent pseudomeningocele, intracranial hypotension, and revision surgery remain important challenges in cases complicated by DO or sagittal imbalance. Emerging technologies such as bioactive hydrogels, 3D-printed scaffolds, nanomaterials, and AI-driven prediction models show promise but require further clinical validation. Continued multicenter collaboration and consistent definitions of CSF leakage will be essential for establishing stronger evidence-based guidelines.

## Figures and Tables

**Table 1:** Incidence and Risk Factors for Dural Tear in Anterior Cervical Spine Surgery.

Category	Key Findings
Procedure Type	Routine ACDF/ACCF: very low incidence (<0.5%)
Pathology	OPLL with dural ossification: 4–32% (up to 63%)
Surgical Approach	Anterior: 31% vs. Posterior: 9.3% (OR 1.9)
Patient Factors	Older age (>65), obesity (BMI ≥ 30), steroid use, revision surgery
Radiological Predictors	Double-layer and hook signs, K-line negativity, OPLL occupying ratio ≥ 60%

**Table 2:** Pathophysiological Mechanisms of Dural Fragility and CSF Leakage.

Mechanism	Description
Cellular Metaplasia	Fibroblast at the PLL–dura junction differentiates into osteogenic cells expressing BMP-2, Runx2, and VEGF
Loss of Elasticity	Collagen disorganization and fragmentation of elastic fibers reduce tensile strength
Biomechanical Stress	Shear strain at ossified junctions exceeds 15%, directly tearing fragile dura
CSF Pressure Effect	Pulsatile CSF waves expand microtears into larger defects, forming a pseudomeningocele
Inflammatory Cascade	TNF-α and IL-1β increase vascular permeability and degrade ECM, delaying closure

**Table 3:** Surgical Repair Techniques for Anterior Cervical Dural Tear.

Defect Size	Recommended Strategy	Materials/Adjuncts	Reported Success
Small (<5 mm)	Gelatin sponge with short-term lumbar drainage	Gelfoam^®^, fibrin glue	90–95%
Moderate (5–10 mm)	Fascia or pericardium onlay reinforced with fibrin or PEG hydrogel	Tisseel^®^, Duraseal^®^	>95%
Large (>10 mm)	Composite “sandwich” repair using artificial dura + sealant + fat graft	DuraGen^®^, TachoSil^®^	>95%
Complex/Recurrent	Vascularized flap + PRVCLD system	SCM or pectoralis flap	100%

**Table 4:** Postoperative Management and Outcomes.

Management Step	Key Practice	Clinical Outcome/Evidence
Pressure Regulation	Pump-regulated lumbar drainage (PRVCLD): 6–8 cm H_2_O, 5–10 mL/h for 3–5 days	90% leak resolution
Mobilization	Early ambulation after drainage < 30 mL/day (48–72 h)	Improved comfort, fewer pulmonary issues
Pseudomeningocele	Observation (<2 cm); aspiration or graft for >4 cm lesions	90% spontaneous resolution
Reoperation Rate	Persistent leak/implant failure/OPLL progression	~5% overall

## Data Availability

Not applicable.

## Abbreviations

The following abbreviations are used in this manuscript:

DT	Dural Tear
CSF	Cerebrospinal Fluid
OPLL	Ossification of Posterior Longitudinal Ligament
ACDF	Anterior Cervical Discectomy and Fusion
ACCF	Anterior Cervical Corpectomy and Fusion
PEG	Polyethylene Glycol
PRVCLD	Pump-Regulated Volumetric Continuous Lumbar Drainage
DO	Dural Ossification
MRI	Magnetic Resonance Imaging
CT	Computed Tomography
PGA	Polyglycolic Acid
SCM	Sternocleidomastoid
PCL	Polycaprolactate
EBP	Epidural Blood Patch
AI	Artificial Intelligence
